# Farmers' Uptake of Animal Health and Welfare Technological Innovations. Implications for Animal Health Policies

**DOI:** 10.3389/fvets.2019.00410

**Published:** 2019-11-20

**Authors:** Jiayi Liu, Luiza Toma, Andrew P. Barnes, Alistair Stott

**Affiliations:** ^1^Biomathematics and Statistics Scotland, Edinburgh, United Kingdom; ^2^Scotland's Rural College, Edinburgh, United Kingdom

**Keywords:** latent class analysis, technology uptake, model selection, farmer typology, animal health

## Abstract

The paper analyses the uptake of animal health and welfare technologies by livestock farmers focusing on the identification of different behavioral patterns occurring in subpopulations of farmers and the assessment of the effect socio-economic and attitudinal factors have on these patterns. The technologies of interest include new genomic technologies, animal electronic identification (EID) for farm management, cattle surveillance, welfare qualitative behavioral assessment, anaerobic digestion, pedometers or activity monitors to detect oestrus and increase fertility/conception, and webcams/smart phones/tablets for animal husbandry. We use latent class analysis modeling and cross-section survey data to construct typologies of farmers based on technological uptake and heterogeneous characteristics. Our results suggest that, while three fifths of the farmers are “non-adopters,” a third is classified as “current adopters” of animal EID for farm management, and a twelfth as “future adopters” of either or more types of animal health and welfare technologies. Age, agricultural income, perceived difficulty to invest in new technologies, agri-environmental scheme membership, and frequency of access to information on animal EID for farm management and cattle surveillance through British Cattle Movement Service, are significant predictors of typology membership. The findings are policy relevant as they give quantitative evidence on the factors influencing technological uptake and, as such, help identify the most likely adopters and optimize the cost of targeting them. As information access was found to be among the factors influencing multiple technology adoption, policy instruments should include the provision of training as regards the implementation of technologies and their combined impact on farm. Farmers' adoption of interrelated innovations suggests the need to coordinate individual policies aimed at encouraging uptake of different technologies. As shown here, this would concern not only synchronizing animal health and welfare policies, but also their interaction with others such as agri-environmental ones. Moreover, the results show that animal health policies requiring regulatory compliance may lead to voluntary uptake of additional or complementary technologies which relate to not just meeting but exceeding standards of animal welfare and health practices.

## Introduction

Farming populations within most countries tend to exhibit a range of goals and farming objectives, reflecting production-orientation and embedding of social values (1–4). This heterogeneity found within farming populations presents particularly intractable problems for policy makers seeking to promote multiple goals for their agricultural systems. Over the last three decades agricultural systems in high income countries have shifted from the aim of solely producing food to one in which environmental and social considerations need to be met. In Europe, these changing policy signals are embodied in the reform documents of the Common Agricultural Policy and related regulations and support focused on socially desirable consequences such as protection and enhancement of animal health, welfare and the environment (4). In response, farmers have tended to exhibit a range of motivations toward these changing goals (2, 5, 6). Based on the psychological concept of social distance, Kagan and Scholz (7) and Braithwaite (8) developed what they term “motivational postures” which range across a variety of levels of engagement with social and regulatory standards. Within the literature on farming populations similar mixtures of motivations have been found with respect to water quality prevention (9, 10); climate change awareness and mitigation (11–14) and the reform of the Common Agricultural Policy (2, 15). The argument used by these authors for exploring and classifying the degree of heterogeneity within a farming population are 2-fold, firstly to understand responses to a possible policy response (2) and secondly to offer direction for apportioning the level of advisory engagement or framing messages pertinent to particular issues within policy (11).

An underexplored area within the literature on farmer typology relates to the uptake of animal health and welfare technologies. This represents a mixture of regulatory compliance (e.g., electronic identification (EID) scheme for sheep), and voluntary standards established by interest groups [e.g., Linking Environment and Farming (LEAF)] or established by processors or retailers to ensure a higher premium for enhanced standards. Hence, the motivation for this study is to explore, using a classification approach and survey data, farmers' motivations for uptake of technologies which relate to meeting and exceeding standards of animal welfare and health practices, their classification in typologies based on technology adoption behavior, and implications for animal health policy.

## Data and Method

### Survey Design and Data Collection

The data used in this study are drawn from a representative telephone survey of Scottish agricultural holdings, which took place in 2013. While the central aim of the survey was to identify the impact the Common Agricultural Policy (CAP) reforms on farm structural changes, a specific section was focused on animal health and welfare technological uptake on Scottish farms. The sampling frame (~10,000 farms) was derived from the June Agricultural Census (JAS) and stratified by region, activity, size and farming enterprise. A potential limitation of the study is related to the JAS under-representation of “very very small” farms (business holdings with <0.5 standard labor requirements). However, based on findings from the literature (16) confirmed by this study, larger farms are more likely to uptake technologies and thus we consider this potential bias to be inconsequential to the results of the analysis. This study analyzed data for 1,746 livestock farms from a total of 2,416 fully completed questionnaires from livestock, crop and mixed farms. After discarding missing values, the total number of valid observations was 1,502.

The section of the questionnaire used in this analysis and consistent with the use of Latent Class Analysis included close-ended questions on the following: socio-economic characteristics (gender, age, education, tenure status, duration of involvement in the business, number of employees, agricultural income, recipient of Single Farm Payment (SFP), succession prospects, organic certification, and participation in agri-environmental schemes); perceived effects on business management from changes in technology, succession planning, access to advice/information, changes in animal welfare regulations and policies; perceived difficulty to invest in new technologies; frequency of access to novel technological information on EID for farm management and cattle surveillance; perceived effects of the use of new knowledge or technology on the welfare of animals on own farm during the past 10 years; technology adoption behavior during the past 10 years (new genomic technologies, animal EID for farm management, cattle surveillance, qualitative behavioral assessment (QBA), anaerobic digestion, pedometers or activity monitors to detect oestrus and increase fertility/conception, webcams/ smartphones/tablets for animal husbandry); and intentions to adopt technologies during the next 10 years (new genomic technologies, animal EID for farm management, cattle surveillance, qualitative behavioral assessment (QBA), anaerobic digestion, pedometers or activity monitors to detect oestrus and increase fertility/conception, webcams/smartphones/tablets for animal husbandry).

The aforementioned statements were used to form explanatory variables (whose descriptive statistics are presented in Table A1) influencing behaviors and intentions to uptake technologies, and independent variables representing behaviors and intentions (whose descriptive statistics are presented in Table 1).

**Table 1:** Descriptive statistics of technology adoption behaviors and intentions.

	**Since 2005 have you applied/started to apply on your business/holding any (technological) innovations:**	**In the next 10 years are you planning to apply on your business/holding any (technological) innovations:**
	**(%) said YES**	**(%) said YES**
New genomic technologies	87 (5.8%)	138 (9.2%)
Animal EID for farm management	447 (29.8%)	354 (23.6%)
Cattle surveillance	199 (13.2%)	230 (15.3%)
QBA	73 (4.9%)	93 (6.2%)
Anaerobic Digestion	37 (2.5%)	86 (5.7%)
Pedometers or activity monitors to detect oestrus and increase fertility/conception	85 (5.7%)	116 (7.7%)
Webcams/smart phones/tablets for animal husbandry	139 (9.3%)	192 (12.8%)

The statistics presented in Table 1 show low rates of adoption and intentions to adopt except for animal EID for farm management (almost a third of the sample) and cattle surveillance (about an eighth). Intentions to uptake showed higher percentages than the current behaviors associated to most technologies, more strongly so for anaerobic digestion (more than twice), genomic technologies (higher by a third) and webcams/smart phones/tablets for animal husbandry (higher by more than a quarter).

### Latent Class Analysis

Latent class analysis (LCA) (17, 18) is a statistical technique for the analysis of multivariate categorical data, also known as a type of finite mixture model. Applied in social sciences, LCA is often used to identify behavioral typologies. Typically, the observed data take the form of a series of categorical responses referred to as manifest variables or items e.g., in this study these are questions about technological uptake and intentions (dichotomous variables). LCA classifies individuals into classes, which are latent when the classification criterion is based on a latent variable (i.e., a construct that is not directly measureable used to estimate the distribution for each subgroup of the population across the items of interest). The latent class (LC) classification model assigns each observation into a latent class with an estimated probability—the latent class membership—which in turn produces expectations about how that observation will respond on each item. Furthermore, the LC classification model is extended using an LC regression model which allows the inclusion of class-specific explanatory variables/covariates to predict latent class membership. This makes LCA the appropriate tool for answering the purpose of this study of identifying typologies of Scottish farmers based on health and welfare technological adoption, and estimating the effect of variables such as socio-economic characteristics to predict the latent class membership.

As regards testing and estimating LC models, the traditional likelihood ratio test (LRT) cannot be used to test nested LC models due to its assumption of a chi-square difference distribution which is not applicable in LCA (19, 20). Therefore, the test of statistical significance of nested models is not easily met and thus a *p*-value is not a straightforward means to comparing nested models. The literature offers alternative likelihood-based techniques, for example Lo et al.'s (21) approximation of the LRT distribution [albeit disputed by (22) who claimed that there was a flaw in their mathematical proof of the test for normally distributed outcomes] or the bootstrap likelihood ratio test (BLRT) by McLachlan and Peel (20). The principle behind BLRT is to use bootstrap samples to estimate the distribution of the log likelihood difference test statistic. Theoretically, the BLRT can therefore provide a *p*-value between a paired comparison of the LC classification models with k-1 and k class solutions. However, implementation of the BLRT has not commonly occurred due to the fact that the paired comparison between two nested models is time consuming, especially when the classification model contains a large number of parameters to estimate. More practical alternatives to the traditional LRT technique include the Akaike's Information Criterion (AIC) (23) and the Bayesian Information Criterion (BIC) (24), which are statistical information criteria (IC) commonly used for the indication of goodness-of-fit and comparison between nested models. Nylund et al. (25) compared the performance of the traditional ICs used to determine the number of classes in mixture type models. They concluded that BIC is superior to all other ICs, especially for larger datasets, and this confirms findings of other authors (18, 26, 27). In contrast, AIC has been shown to overestimate the correct number of components in finite mixture models (28, 29).

Thus, in this study, we used BIC to determine the number of latent classes in each of the LC classification models estimated and as a criterion for model selection among the nested LC regression models (with class-specific covariates). We used backward elimination technique for model selection of the nested LC regression models, where the full model was initially set up to include all covariates of interest and then step by step variables whose absence improves model fit (iteratively testing for the smallest BIC value) were removed until no further improvement was possible.

The LC models were fitted using the package poLCA in the statistical software R (30, 31). poLCA is an R package used to estimate LC classification models for manifest variables with any number of possible outcomes, and LC regression models with class-specific covariates.

## Results

The aforementioned methodological steps were applied to the study of the current adoption and intentions to adopt seven types of animal health and welfare innovations (presented in Table 1). The analysis followed two stages: firstly, it identified the possible number of latent classes from various LC classification models based on technological innovation adoption and intentions to adopt. Namely it identified different characteristics from individuals' patterns of response as regards both current and intended uptake, which led to the formation of subgroups (latent classes) in the population. In the second stage it examined the effects of the explanatory variables of interest on the latent class membership. This is an essential step which explains which factors can predict individuals' latent class membership.

### Three-Class LC Classification Model

#### Item Elimination

Farmers were asked two questions, one about their current technological uptake behavior and another about their intentions, both applied for each of the seven technologies. The 14 questions (items) were used to identify the latent classes in the LC classification model. However, responses on current adoption of five out of seven technologies in all LC classification models had very low (close to zero) estimated probabilities across all latent classes, except for the uptake of animal EID for farm management and cattle surveillance. Thus the final LC classification model included nine items: two items of current adoption (animal EID and cattle surveillance) and all seven items based on intentions to uptake. The statistical results presented in the remaining of this paper consider only nine items.

#### Determining the Number of Latent Classes

Latent class classification models from two-class to five-class solution were estimated. Table 2 shows BIC and AIC values for LC models with two-class, three-class, four-class and five-class solutions.

**Table 2:** BIC and AIC for LC classification models with two-class to five-class solutions.

**LCA model**	**BIC**	**AIC**
2-class LCA	8695.37	8572.25
3-class LCA	8476.23	8288.87
4-class LCA	8451.47	8199.87
5-class LCA	8455.72	8139.89

BIC suggested the selection of the LC model with four-class solution, as this model reached the minimum value (8451.47). As expected, AIC tends to over-fit the data, which means that AIC values decreased while the number of latent classes increased.

Next we checked graphically the characteristics of each latent class from the LC classification model with four-class solution (Figure A1) and some issues were identified. Namely there was an equal probability of answering “yes” or “no” to certain items in certain latent classes. This was the case for EID uptake in latent classes three and four, and cattle surveillance uptake in latent class four. This issue is referred to as unidentified item in the study of LCA. It is important in an LC classification model that all class-memberships in each latent class are identified, i.e., the probability of being in one response category should be significantly >0.5. Thus we discarded the four-class solution model and the preferred model was the LC model with a three-class solution.

**Figure 1:**
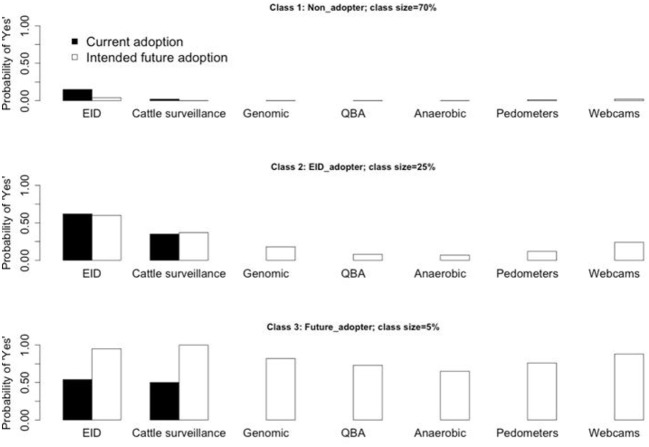
The characteristics of the LC classification model with three-class solution.

The characteristics of each identified subgroup of farmers from the LCA three-class solution model are shown in Figure 1. The majority of farmers (70%) were classified in the first class. This class represented a subgroup of farmers who are technological “non-adopters,” with small probabilities (<0.2) of saying “yes” to both uptake and intentions to uptake animal health and welfare technologies. The second class contained one quarter of the sample of farmers who had a higher probability (about 0.6) of saying “yes” to both uptake and intentions to uptake animal EID for farm management. Therefore, the second latent class was labeled as the “EID adopters.” Finally, the third latent class contained only about 5% of the farmers who have greater probabilities (values between 0.65 and 1.00) of saying “yes” to intentions to uptake animal health and welfare technological innovations. The third class therefore represented the future technology adopters, which was labeled as the “future adopters.”

### Three-Class LC Classification Model With Explanatory Variables

We tested the effect of explanatory variables of interest (see Table A1) on latent class membership. Model selection between LC classification models with a large number of explanatory variables is computationally demanding and disentangling dependency among explanatory variables is not always straightforward. Therefore, we applied backward selection (32) based on BIC to estimate an LC multiple regression model. For nested models, a model with a smaller BIC value is an indication of improved goodness-of-fit.

The final model is presented in Table 3, which shows that six variables (age; intention to remain in agri-environmental schemes until 2020; perceived difficulty of investing in new technologies; frequency of access to information on EID for farm management; frequency of access to information on cattle surveillance) out of the 21 variables were significant. Together they predicted individuals' latent class membership.

**Table 3:** The final three-class LC regression model (coefficients are estimated of logarithm of odds ratio using backward model selection technique).

**Model**		**Class 2 vs. Class 1**			**Class 3 vs. Class 1**			**Class 3 vs. Class 2**		
		**Coe**.	**SE**	***P*-value**	**Coe**.	**SE**	***P*-value**	**Coe**.	**SE**	***P*-value**
Intercept		−1.43	0.534	0.008	−2.77	0.807	0.001	−1.34	0.869	0.123
Age		−0.02	0.007	0.005	−0.04	0.010	0.001	−0.02	0.011	0.154
Remain in agri-environmental scheme until 2020: no (vs. yes)		−0.80	0.158	<0.001	−0.76	0.244	0.002	0.04	0.253	0.875
Percentage of agricultural income in total income: (vs. <25%)	25–75%	0.23	0.249	0.363	−0.25	0.379	0.510	−0.48	0.407	0.243
	>75%	0.76	0.222	0.001	0.37	0.332	0.260	−0.39	0.362	0.284
How difficult do you find investing in new technologies?	0.27	0.070	<0.001	0.45	0.111	<0.001	0.18	0.117	0.116	
How often do you look for information on EID for farm management? (vs. never)	weekly	1.75	0.259	<0.001	0.60	0.342	0.079	−1.15	0.367	0.002
	monthly	1.02	0.229	<0.001	0.16	0.325	0.612	−0.85	0.357	0.017
	yearly	1.34	0.279	<0.001	0.55	0.416	0.182	−0.79	0.436	0.071
How often do you look for information on cattle surveillance through British Cattle Movement Service? (vs. never)	weekly	0.28	0.234	0.228	2.18	0.464	<0.001	1.89	0.481	<0.001
	monthly	0.63	0.217	0.004	2.12	0.445	<0.001	1.49	0.465	0.001
	yearly	−0.14	0.325	0.676	1.07	0.584	0.066	1.21	0.625	0.053

We recoded age (initially a variable with five categories, see Table 1) based on the assumption that the effect of age on the individuals' latent class membership was linear. We set values of 30, 40, 50, 60, and 70 to represent the average age for each age group, respectively, and examined the effect of age in increments of 10 years on individual's class membership.

We also recoded the variable “proportion of agricultural income in total income from this business/holding” (initially with five categories) into a variable with three categories (Table 3) due to the fact that more than half of the farmers stated that more than 75% of their income was from agriculture. The three recoded categories represented the group with low proportion of agriculture income (<25%, this being the reference group to which the other two categories were compared), the group with mixed type of income (25–75%) and the group with mostly agricultural income (more than 75%).

Additionally we recoded the variable “perceived difficulty of investing in new technologies” (initially a variable with five categories) into a numerical variable based on the assumption that the equal distance between each paired categories was not fundamental to the focus of this study.

**Figure 2:**
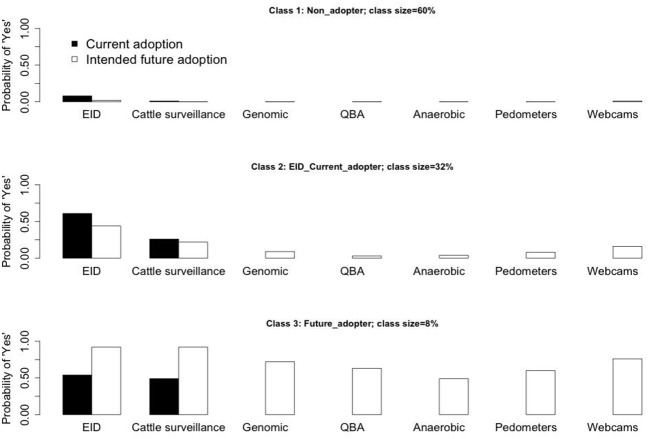
The characteristics of the LC three-class regression model (with six explanatory variables).

Following results presented in Table 3, further clarification of two issues was needed for a better understanding of the results. The proportions of estimated class membership shifted to some extent compared to the initial three-class LC classification model. The characteristics of the three latent classes told a similar story but with a slight diversion (see Figure 2).

Figure 2 presents the characteristics of the three latent classes identified in the LC classification model, which show a variation after the inclusion of the explanatory variables. The first class contained 60% (previously 70%) of the farmers, but still with very low probabilities for both uptake and intentions to uptake technologies. Therefore, the first class still represented the “non-adopters.” The second class contained about one third (previously 25%) of the farmers, but the probability of the intentions to uptake EID dropped below 0.5 (0.44). Since the probability of current adoption of EID was still >0.5 (0.61), the results suggested the second class became the group of “EID current adopters.”

The third class contained 8% (previously 5%) of the population, and it was still referred to as the “future adopters.”

The estimated coefficients presented in Table 3 are logarithms of odds ratios as the latent class analysis presents the probability of preferring “yes” over “no” (odds ratio) then takes a natural logarithm of the odds ratio. Additionally, the estimated coefficients are presented as paired comparisons between two latent classes to the effect of the logarithm of odds ratio. This leads to a less than straightforward interpretation of the coefficients. The rule of thumb is that for a categorical variable a positive coefficient implies that the comparator latent class has greater logarithm of odds value than the base latent class while moving from the reference category to the comparator category of this categorical variable. Thus the practical interpretation is that a positive coefficient implies an increasing likelihood of belonging to the comparator latent class group (if “yes” rather than “no” was stated) when the comparator category rather than the reference category of this categorical variable was chosen. On the other hand, a negative coefficient implies an increasing likelihood of belonging to the base latent class group (if “yes” rather than “no” was stated) when the comparator category rather than the reference category of this categorical variable was chosen. For a continuous covariate, a positive coefficient implies an increasing likelihood of belonging to the comparator latent class group while increasing the value of the variable, and the opposite holds, namely a negative coefficient implies an increasing likelihood of belonging to the base latent class group while increasing the value of the variable.

**Figure 3:**
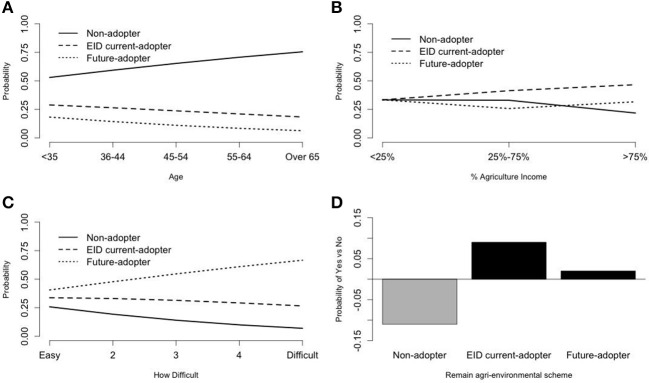
**(A)** Age as predictor of class membership based on technological uptake and intentions. **(B)** Agricultural income as predictor of class membership based on technological uptake and intentions. **(C)** Difficulty to invest in new technologies as predictor of class membership based on technological uptake and intentions. **(D)** Agri-environmental scheme membership as predictor of class membership based on technological uptake and intentions.

**Figure 4:**
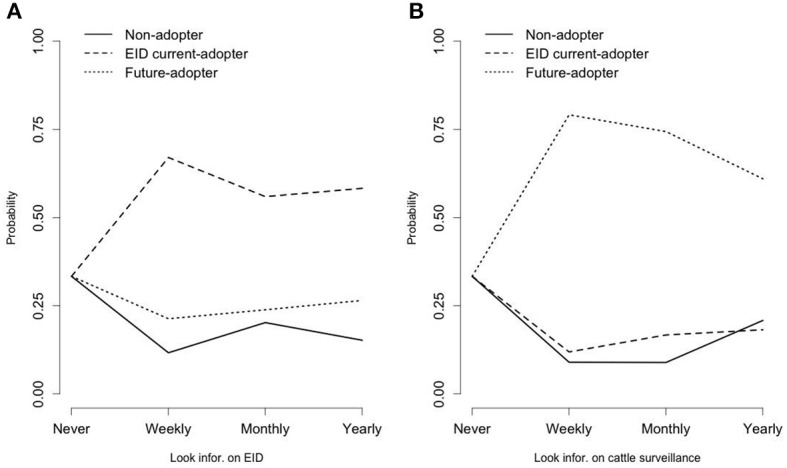
**(A)** Frequency of access to information on EID for farm management as predictor of class membership based on technological uptake and intentions. **(B)** Frequency of access to information on cattle surveillance as predictor of class membership based on technological uptake and intentions.

Still it would be more straightforward to visualize how each of the six covariates can predict the probability of latent class memberships while changing each of their outcomes. Therefore we used Figures 3, 4 to graphically represent the estimated effects of the six covariates presented in Table 3. The estimated probability of latent class membership was computed without the intercepts (to remove the effect due to different latent class group size), which enabled us to see the pure effect of each covariate.

To begin with, the effect of age is presented in Figure 3A. It shows that with increasing age there is an increasing probability of becoming a “non-adopter.” This is in contrast with the other two classes, where the probabilities decline with increasing age, indicating that younger farmers have higher probabilities to become either “EID current adopters” (class two) or “future adopters” (class three) than older farmers.

The proportion of agricultural income in the total income had also a significant effect on the latent class membership (Figure 3B). Farmers are more likely to become “EID current adopters” if a large proportion (higher than 75%) of their income was from agriculture on farm when compared with farmers with lower agricultural income (<25%). In other words, famers who stated that their farms have <25% agricultural income are more likely to become the “non-adopters.” There is no statistical evidence for a significant association between the proportion of agricultural income and the membership of the “future adopters” class, although graphically the dotted line (future adopters) catches up with the dashed line when the proportion of agricultural income is >75%.

Figure 3C presents the effect of the variable “perceived difficulty of investing in new technologies” on latent class membership. Both the “EID adopters” and the “future adopters” groups had greater probabilities than the “non-adopters” group, which confirms the positive logarithms of odds ratios in Table 3 (0.27, 0.45). Moreover, farmers with stronger perceptions as regards the difficulty of investing in new technologies showed a higher probability of belonging to the “future adopters” group. Although there was a mild declining trend in the “EID current adopters” group, the odds over the “non-adopters” group were still greater than one.

The “non-adopters” group showed a strong declining pattern positively associated with stronger perceptions as regards the difficulty of investing in new technologies. In other words, farmers who found it more difficult to invest in new technologies were more likely to become either “EID current adopters” or “future adopters.”

Figure 3D shows the effect of the agri-environmental scheme membership on the probability of belonging to one of the three latent classes. The pattern suggests that current members of agri-environmental schemes who were more likely to cease membership by 2020 were also more likely to belong to the “non-adopters” group compared with farmers with an agri-environmental scheme membership who were more likely to belong to either the “EID adopters” or the “future adopters” groups.

The last two of the explanatory variables, frequency of access to information on EID for farm management and cattle surveillance, are presented in Figures 4A,B. Figure 4A shows a pattern which suggests that more informed farmers (who checked information about EID more frequently) had a higher probability of belonging to the “EID current adopters” group than those who never looked for such information. However there is no significant association between the frequency of looking for information and the likelihood of becoming “future adopters.”

The pattern in Figure 4B about farmers' frequency of access to cattle surveillance information shows that farmers who looked for cattle surveillance information (especially on a weekly or monthly basis) had a much higher probability to become “future-adopters” than those who never looked for such information.

## Discussion and Conclusions

This study identified three groups of farmers in a typology analysis based on farmers' uptake and intentions to uptake animal health and welfare technologies. The characteristics of the three groups were estimated with and without controlling for socio-economic and attitudinal covariates.

When no explanatory variables were considered, the majority of farmers (more than two thirds) were classified as “non-adopters,” i.e., farmers less likely to uptake or to intend to uptake either or more of the seven types of animal health and welfare technologies analyzed. The second largest group (a quarter of farmers) contained the “EID adopters,” i.e., the farmers already using animal EID for farm management and those willing to uptake animal EID for farm management in the following 10 years. The third and smallest group (a twentieth of farmers) contained the “future-adopters,” i.e., the farmers willing to uptake either or more of the animal health and welfare technologies.

After controlling for socio-economic and attitudinal covariates, the characteristics of the three groups based on technological uptake remained similar for both the “non-adopters” and the “future-adopters,” albeit with a change in size, i.e., the “non-adopters” group decreased to three fifths of farmers, while the “future adopters” group increased to include about a twelfth of farmers. However a more significant change occurred in the second group labeled “EID adopters” in the model without covariates (which contained farmers already uptaking or willing to uptake animal EID for farm management in the next 10 years), which after controlling for covariates became the “EID current adopters” group (which contained farmers showing current uptake).

The effects of the six class-specific explanatory variables included in the three-class latent class regression model showed expected patterns that confirmed findings from the literature.

Age can be a significant influence on technological uptake in many technology adoption studies (33, 34). Our results show that the younger the farmers, the more likely they were to belong to either the group of “EID current adopters” or to the “future-adopters” group. On the other hand, the older the farmers, the more likely they were to be part of the “non-adopters” group.

Farmers' financial status (income, investment, profitability) has been found to influence technological adoption (34–37). Deriving relatively more income from agricultural activities and thus demonstrating a stronger focus on agricultural rather than non-farm activities is more strongly linked to adoption of technologies directly connected with agricultural production (38). Our results support the latter and suggest that farmers with a larger proportion (>75%) of their total income originating from agriculture were more likely to belong to the group of “EID current adopters.” On the other hand, farmers with <25% agricultural income were more likely to belong to the “non-adopters” group.

Farmers' perceptions of the difficulty to invest in new technologies influenced their membership in a specific technological uptake group, namely those with stronger perceptions about investment difficulties were more likely to belong to the “EID current adopters” group or to be willing to become the “future adopters.” This finding might be explained by the fact that farmers who have adopted technologies or intended to adopt were more aware of the investment needs related to technological uptake and might have experienced investment difficulties while uptaking or attempting to uptake new technologies. Another potential reasoning could be linked to the size of investment required for the specific case of EID technology uptake, which is less significant than that required for uptake of some of the other technologies mentioned.

The literature has shown that innovative behaviors tend to go hand in hand, i.e., individuals who adopted specific innovations are also more likely to uptake or intend to uptake other innovations more or less related to the ones adopted in the past (38–40). Our results showed a positive relationship between membership in agri-environmental schemes and uptake of animal health and welfare technological innovations. Farmers who were members of agri-environmental schemes and who intended to maintain their membership during the next 10 years were more likely to belong to either the “EID current adopters” or the “future adopters” groups.

And finally, one of the main influences on technological uptake, access to information about the specific technologies has been consistently referred to in the technology adoption literature (41–47). Our results suggest that the higher the frequency of access to information on animal EID for farm management, the higher the probability of farmers belonging to the “EID current adopters” group. Similarly, farmers who looked for information on cattle surveillance through British Cattle Movement Service on a weekly or monthly basis were more likely to become the “future adopters” than those who never looked for information.

The findings are policy relevant as they give quantitative evidence on the factors influencing technological uptake and, as such, help identify the most likely adopters and optimize the cost of targeting them. As information access was found to be among the factors influencing multiple technology adoption, policy instruments should include the provision of training as regards the implementation of technologies and their combined impact on farm. Farmers' adoption of interconnected technological innovations suggests the need to coordinate individual policies aimed at encouraging uptake of different technologies. As shown here, this would concern not only synchronizing animal health and welfare policies, but also their interaction with others such as agri-environmental ones. Moreover, the results show that animal health policies requiring regulatory compliance may lead to voluntary uptake of additional or complementary technologies which relate to not just meeting but exceeding standards of animal welfare and health practices.

## Data Availability Statement

The datasets for this manuscript are not publicly available because confidentiality agreements restrict access to this dataset. Requests to access the datasets should be directed to LT, luiza.toma@sruc.ac.uk.

## Author Contributions

JL led the analysis and contributed to the writing. LT led the writing and contributed to the analysis (variable and model selection). AB and AS contributed to the writing.

### Conflict of Interest

The authors declare that the research was conducted in the absence of any commercial or financial relationships that could be construed as a potential conflict of interest.
