# Supplementary material for: Farmers' Uptake of Animal Health and Welfare Technological Innovations. Implications for Animal Health Policies
Source: Front Vet Sci. 2019 Nov 20;6:410. doi: 10.3389/fvets.2019.00410 (PMC6879451; doi:10.3389/fvets.2019.00410)

Appendix

Table A1 Descriptive statistics of explanatory variables of interests (frequency and proportion)

Variable of interest	Outcome	Frequency (%)
Gender	M:	1,212 (80.7%)
	F:	290 (19.3)
Age	<35:	79 (5.3%)
	36-44:	156 (10.4%)
	45-54:	425 (28.3%)
	55-64:	466 (31.0%)
	Over 65:	376 (25.0%)
Educational level	School:	751 (50.0%)
	College:	486 (32.4%)
	Uni/Higher:	265 (17.6%)
Tenure status	Tenant:	362 (24.1%)
	Tenant&Owner:	190 (12.6%)
	Owner:	895 (59.6%)
	Manager:	55 (3.7%)
Number of years of having been involved in the business/holding?	<5:	76 (5.1%)
	[5-10]:	87 (5.8%)
	(10-20):	227 (15.1%)
	>20:	1,112 (74.0%)
Number of people employed on this land	None:	737 (49.1%)
	1-3:	649 (43.2%)
	4 or more:	116 (7.7%)
Until 2020 are you planning to remain/become involved in agri-environmental schemes?	Yes:	623 (41.5%)
	No:	879 (58.5%)
Are you certified organic?	Yes:	80 (5.3%)
	No:	1,407 (93.7%)
	Conversion:	15 (1%)

Are you full time/part time/hobby farmer/other?	FT:	977 (65.0%)
	PT:	330 (22.0%)
	Hobby:	106 (7.1%)
	Other:	89 (5.9%)
Have you identified a potential successor who will eventually take over the management of your farm business?	No:	332 (22.1%)
	Unsure:	426 (28.4%)
	Yes:	744 (49.5%)
How much of your total income from this business/holding is from agriculture?	0%:	100 (6.7%)
	(0-25%):	183 (12.2%)
	[25%-50%]:	209 (13.9%)
	(50-75%):	232 (15.4%)
	>75%:	778 (51.8%)
Since 2005 have any of the following changed the way you manage your business/holdings?		
Changes in technology	No:	682 (45.4%)
	Slightly:	554 (36.9%)
	Significantly:	266 (17.7%)
Succession planning	No:	1,199 (79.8%)
	Slightly:	243 (16.2%)
	Significantly:	60 (4.0%)
Access to advice/information on new opportunities	No:	1,028 (68.4%)
	Slightly:	350 (23.3%)
	Significantly:	124 (8.3%)
Changes in animal welfare regulations and policies	No:	777 (51.7%)
	Slightly:	463 (30.8%)
	Significantly:	262 (17.5%)
How difficult do you find investing in new technologies? (from easy to difficult)	1=easy:	347 (23.1%)
	2:	310 (20.6%)
	3:	514 (34.2%)

	4:	262 (17.5%)
	5=difficult:	69 (4.6%)
Do you receive SFP?	No:	281 (18.7%)
	Yes:	1,221 (81.3%)
How often do you look for information on EID for farm management?	Never:	481 (32.0%)
	Weekly:	331 (22.0%)
	Monthly:	485 (32.3%)
	Yearly:	305 (13.7%)
How often do you look for information on cattle surveillance through British Cattle Movement Service?	Never:	506 (33.7%)
	Weekly:	376 (25.0%)
	Monthly:	500 (33.3%)
	Yearly:	120 (8.0%)
How much do you agree that since 2005 you have improved the welfare of your animals through new knowledge or technology? (strongly disagree to strongly agree)	1=Strongly Disagree:	22 (1.5%)
	2=Disagree:	194 (12.9%)
	3=Neither:	140 (9.3%)
	4=Agree:	9 09 (60.5%)
	5=Strongly Agree:	237 (15.8%)

Fig A1 The characteristics of latent classes of the LC classification model with four-class solution

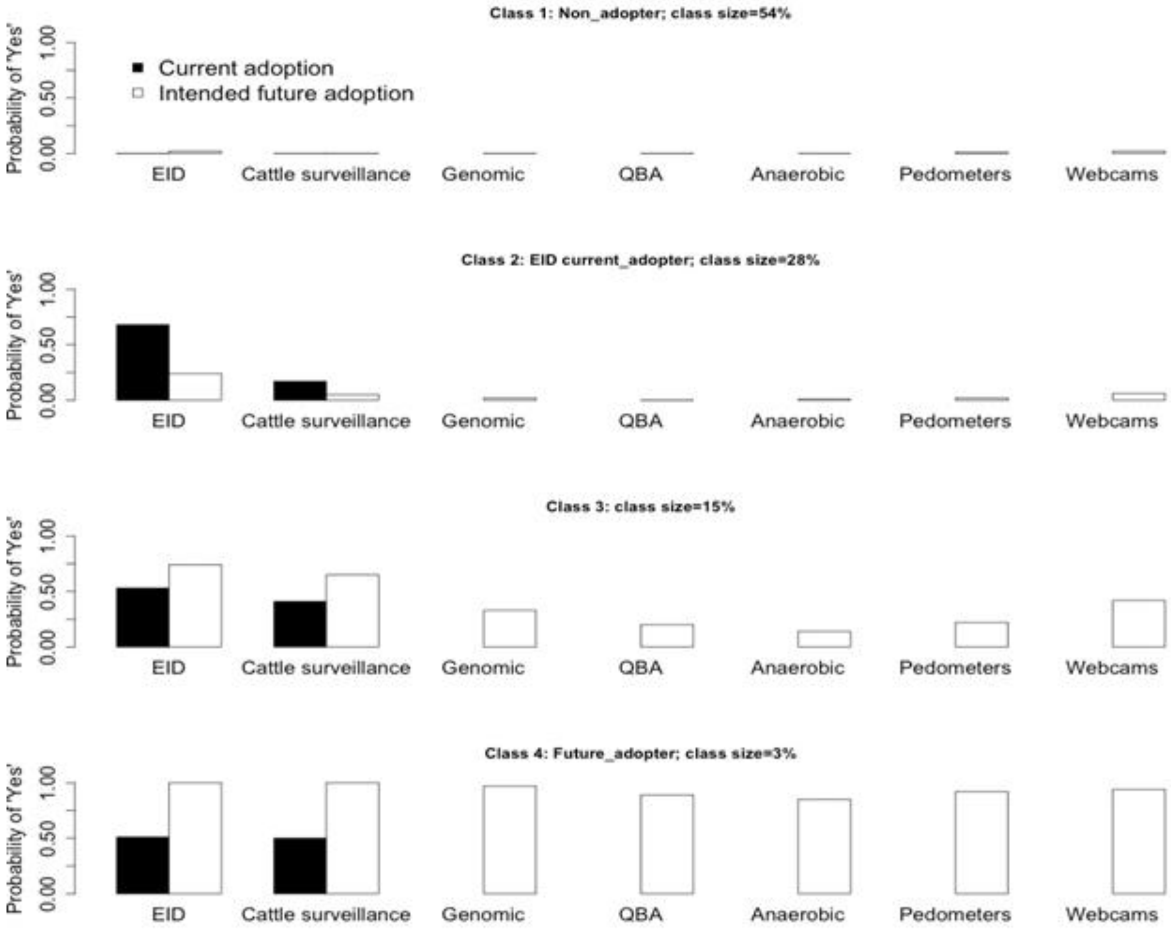

Supplement: Supplementary file 1 [file Data_Sheet_1.pdf]
